# The Quality Initiative in Rectal Cancer (QIRC) trial: study protocol of a cluster randomized controlled trial in surgery

**DOI:** 10.1186/1471-2482-8-4

**Published:** 2008-02-15

**Authors:** Marko Simunovic, Charles Goldsmith, Lehana Thabane, Robin McLeod, Franco DeNardi, Timothy J Whelan, Mark N Levine

**Affiliations:** 1 Department of Surgery, McMaster University, St. Joseph's Healthcare, G838-50 Charlton Ave. East, Hamilton, Ontario, L8N 4A6, Canada; 2 Department of Clinical Epidemiology and Biostatistics, Faculty of Health Sciences, McMaster University, 1200 Main St. W., Hamilton, Ontario, L8N 3Z5, Canada; 3 Juravinski Cancer Centre, 699 Concession St., Hamilton, Ontario, L8V 5C2, Canada; 4 Centre for Evaluation of Medicines, Biostatistics Unit, St. Joseph's Healthcare, 50 Charlton Avenue East, 3rd Floor Martha, Room H325, Hamilton, Ontario, L8N 4A6, Canada; 5 Division of General Surgery, Mount Sinai Hospital, 600 University Avenue, Toronto, Ontario, M5G 1X5, Canada; 6 Department of Pathology and Molecular Medicine, McMaster University, Henderson General Hospital, 711 Concession Street, Hamilton, Ontario, L8V 1C3, Canada; 7 Department of Oncology, Faculty of Health Sciences, McMaster University, 1200 Main St. W., Hamilton, Ontario, L8N 3Z5, Canada

## Abstract

**Background:**

Two unfortunate outcomes for patients treated surgically for rectal cancer are placement of a permanent colostomy and local tumor recurrence. Total mesorectal excision is a new technique for rectal cancer surgery that can lead to improved patient outcomes. We describe a cluster randomized controlled trial that is testing if the above patient outcomes can be improved through a knowledge translation strategy called the Quality Initiative in Rectal Cancer (QIRC) strategy. The strategy is designed to optimize the use of total mesorectal excision techniques.

**Methods and Design:**

Hospitals were randomized to the QIRC strategy (experimental group) versus normal practice environment (control group). Participating hospitals, and the respective surgeon group operating in them, are from Ontario, Canada and have an annual procedure volume for major rectal cancer resections of 15 or greater. Patients were eligible if they underwent major rectal surgery for a diagnosis of primary rectal cancer. The surgeon-directed QIRC interventions included a workshop, use of opinion leaders, operative demonstrations, a post-operative questionnaire, and, audit and feedback. For an operative demonstration participating surgeons invited a study team surgeon to assist them with a case of rectal cancer surgery. The intent was to demonstrate total mesorectal excision techniques. Control arm surgeons received no intervention. Sample size calculations were two-sided, considered the clustering of data at the hospital level, and were driven by requirements for the outcome local recurrence. To detect an improvement in local recurrence from 20% to 8% with confidence we required 16 hospitals and 672 patients – 8 hospitals and 336 patients in each arm. Outcomes data are collected via chart review for at least 30 months after surgery. Analyses will use an intention-to-treat principle and will consider the clustering of data. Data collection will be complete by the end of 2007.

**Discussion:**

Lower rates of permanent colostomy and local tumour recurrence in the intervention arm would suggest the QIRC strategy is efficacious. The strategy may act as a template for efforts to improve surgical quality in other areas and will contribute to knowledge on influencing surgeon practice.

**Trial registration:**

Current Controlled Trials ISRCTN78363167

## Background

Surgical resection of rectal cancer is the cornerstone of curative therapy. Typically during rectal cancer surgery the tumour and a contiguous segment of normal bowel are removed and the bowel tract reestablished. For surgically treated patients two unfortunate outcomes are permanent colostomy and local tumor recurrence. For various reasons the operating surgeon may deem it necessary to remove the rectum and anus rendering the patient dependent on a permanent colostomy. Local tumor recurrence is defined as tumour that recurs in the pelvis near the previous operative site [1-3]. It is especially feared since this outcome is usually inoperable and patients, as a result, can suffer a slow, painful death. This study is testing if these two important outcomes, rates of permanent colostomy and local tumour recurrence, can be improved at the hospital level using the surgeon-directed Quality Initiative in Rectal Cancer (QIRC) strategy.

Studies on rectal cancer surgery outcomes usually show that rates of permanent colostomy, local tumour recurrence, and even patient survival vary markedly at the surgeon, hospital, or region level. For example, among the regions of Ontario, Canada researchers showed that rates of permanent colostomy following rectal surgery varied from 31% to 41% [4]. A study examining local tumour recurrence among surgeons operating in five hospitals in Edmonton, Alberta showed that rates varied from 10% to 45% based on surgeon specialty training and procedure volume [5]. These Canadian results are similar to studies in Europe [6-8]. The presence of important outcome variations suggests variation in the quality of delivered surgery.

Total mesorectal excision is a refinement of traditional surgical techniques that stresses sharp dissection of the mesorectum – the lymph node-bearing portion of the rectum – with careful autonomic nerve preservation [9-11]. There are a growing number of single institution series describing improvements in outcomes with the introduction of total mesorectal excision- in particular local tumour recurrence rates as low as 5% and permanent colostomy rates of 10–15% [12-18]. Population based studies from Europe also detail positive changes when surgeons in large areas adopt total mesorectal excision principles [19,20].

Knowledge translation research has identified interventions that may encourage physician behaviour change such as continuing medical education (e.g., workshops), use of opinion leaders, and audit and feedback [21-28]. It is also suggested that multiple interventions are more effective than single interventions. As well, behaviour change may be enhanced by using quality improvement principles such as a participatory and supportive approach that focuses on the system, not individuals; breaking processes down into definable steps that are more readily targeted for improvement; and, decreasing variation in process steps resulting in improved overall quality [29-32]. The QIRC strategy integrated such knowledge translation interventions and quality improvement concepts in an attempt to ensure that hospitals (i.e., the surgeons in the respective hospital) delivered optimal total mesorectal excision-type surgery to patients.

The purpose of this paper is to describe the methodology of our cluster randomized controlled QIRC trial, which is testing if the surgeon-directed QIRC strategy can improve patient outcomes at the hospital (i.e., cluster) level. We used a cluster design to minimize the chances of contamination at the patient and surgeon level. We surmised that patient-level randomization would not work since surgeons exposed to new information or techniques, such as those promoted through the QIRC strategy, would accept or reject such information or techniques for all of their subsequent patients. We surmised that surgeon-level randomization would not work since surgeons in a given hospital often share operative experiences through discussion or direct observation, and thus new information or techniques would likely be shared among surgeons in a given hospital. Since surgeons in Ontario rarely perform rectal cancer surgery at more than one hospital, we were confident that hospital-level cluster randomization would minimize the chances of contamination between the two arms of the QIRC trial.

## Methods and Design

### Study design

This study is a cluster randomized controlled trial with hospitals being the clusters. Hospitals were randomized to the QIRC strategy (intervention group) versus normal practice environment (control group). The QIRC strategy is directed at surgeons in hospitals randomized to the intervention group using a 1:1 allocation ratio.

### Participants

#### Hospitals

**Figure 1:**
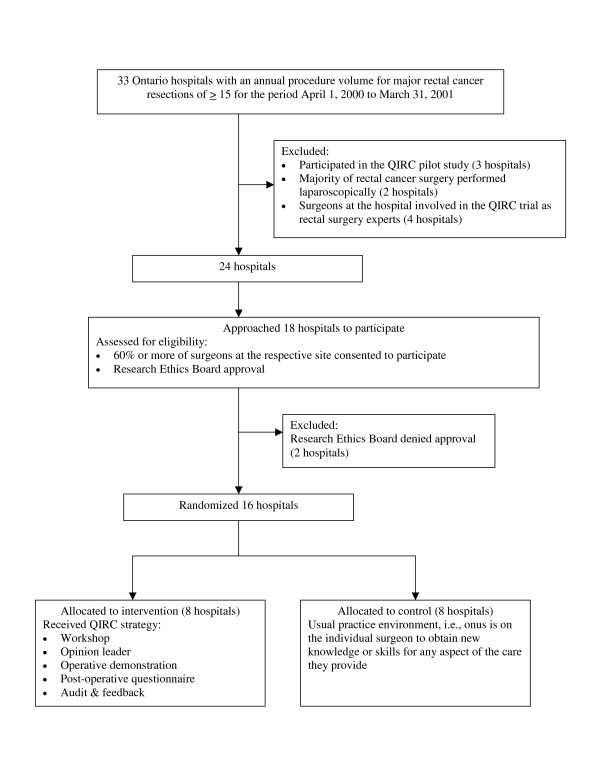
QIRC trial schema.

To avoid targeting sites with minimal procedure volumes, we stated that participating hospitals in Ontario, and the respective surgeon group operating in them, must have an annual procedure volume for major rectal cancer resections of 15 or greater. We identified 33 such hospitals using administrative data for the period April 1, 2000 to March 31, 2001. Hospitals were then excluded if they had participated in the QIRC pilot study [33] (3 sites), if the majority of rectal surgery was performed using laparoscopic techniques (2 sites), or if surgeons at the site were involved in the QIRC trial as rectal surgery experts (4 sites). These two latter criteria were needed since a key intervention of the QIRC strategy was operative demonstrations delivered by rectal surgery experts using open versus laparoscopic techniques (See Interventions below). Nine hospitals were excluded leaving 24 potential sites. Hospitals were then eligible if 60% or more of surgeons at the respective site consented to participate and if the respective research ethics board approved the study. Sample size calculations indicated the need for 16 sites. For the first 18 hospitals approached, nearly every surgeon consented to participate and 16 hospital ethics boards approved the study protocol. (Figure 1)

#### Patients

Patients were eligible if they underwent major rectal cancer surgery (i.e., partial or complete segmental resection of rectum with or without an anastamosis) for a diagnosis of primary rectal cancer. Thus patients who received local excisions were not eligible. We defined a rectal versus colon cancer as a tumour located within 15 cm of the anal verge by rigid sigmoidoscopy, or, a tumour at or below the level of the sacral promontory as seen at the time of surgery. These anatomic landmarks ensured the inclusion of patients that would potentially benefit from total mesorectal excision -type surgery. Tumour stage was not used as an exclusion criterion since issues of permanent colostomy and local recurrence are relevant to all patients undergoing surgery.

### Intervention

The QIRC strategy is a set of interventions directed at surgeons working in a given hospital. The interventions include: a workshop, use of opinion leaders, operative demonstrations, a post-operative questionnaire, as well as audit and feedback.

#### Workshops

These were used to introduce to participating surgeons all aspects of the study and new information. For the latter, topics included rectal cancer surgery outcomes in Ontario and elsewhere, total mesorectal excision, and, knowledge translation and quality improvement. The intent of the workshop was to promote thought in three areas: 1) There is a need to improve population level rectal cancer surgery outcomes in most jurisdictions. 2) The QIRC strategy may help surgeons improve these outcomes. 3) The pursuit of surgical excellence is an active and continuous process.

#### Opinion leaders

At each workshop, participating surgeons selected an opinion leader for their hospital using a validated approach [34]. In summary, the selection was based on the opinion leader having three attributes including a high level of clinical expertise, a willingness to share knowledge, and being educationally influential. The opinion leader acted as a local resource person on issues pertinent to the study. For example, the opinion leader encouraged their colleagues to participate in operative demonstrations.

#### Operative demonstration

Participating surgeons invited a study team surgeon to assist them with a case of rectal cancer surgery. The main intent was to demonstrate total mesorectal excision techniques. Surgeons were encouraged to participate in a minimum of two operative demonstrations. The participating surgeon retained full control over all peri-operative and operative decision-making.

#### Operative questionnaire

This questionnaire was completed after each rectal cancer surgery. The questions were designed to prompt surgeons to re-examine key total mesorectal excision operative steps [see Additional file 1].

#### Audit and feedback

Data (e.g., rates of permanent colostomy) were provided to individual surgeons and in a group forum for overall hospital results. The intent of feedback was to encourage individual surgeons to constantly self-examine surgical decision-making.

### Control arm

Hospitals in the control group represented the normal environment where the onus is on the individual surgeon to obtain new knowledge or skills for any aspect of the care they provide. Efforts to improve the quality of rectal cancer care among control arm surgeons were not facilitated by the study team.

### Objectives

#### Primary objective

We wish to test the efficacy of the QIRC strategy to improve hospital rates of permanent colostomy and local tumour recurrence among patients surgically treated for rectal cancer. We hypothesize that hospitals exposed to the QIRC strategy (intervention) will have better outcomes – lower rates of permanent colostomy and local tumour recurrence – compared to hospitals in the normal practice environment (control).

#### Secondary objective

We wish to test if the QIRC strategy can lead to improved quality of life, and improved bladder, bowel, and sexual function among patients surgically treated for rectal cancer. We hypothesize that patients treated in hospitals exposed to the QIRC strategy (intervention) will have higher scores on quality of life measures, and bowel, bladder and sexual function questionnaires compared to patients treated in control arm hospitals.

### Outcome measures

#### Primary outcomes

Since the hospital is our unit of analysis it is imperative that consecutive patients be included to prevent surgeon selection bias (e.g., excluding patients with difficult tumours perceived as high-risk for negative outcomes). Three methods of patient accrual were used to ensure consecutive patient accrual for primary outcomes – regular (weekly) phone calls to surgeons' offices, review of operating room booking logs, and hospital health records queries. Patient consent was not obtained for primary outcomes since data were collected via retrospective chart review and involved no patient contact. Primary outcomes included:

#### Hospital rate of permanent colostomy

It is usually known at the time of rectal cancer surgery if a person has received a permanent colostomy. However, in cases where a patient receives a temporary stoma 18 months will be allowed for temporary stoma closure, after which time the patient will be designated as having a permanent colostomy. An anastomosis of the rectum must also be functioning for at least three months to be considered a non-permanent colostomy. This will prevent a positive outcome being marked for a patient who subsequently receives a colostomy for problems such as an anastomotic leak or severe incontinence.

#### Hospital rate of local recurrence

Local tumor recurrence is usually defined as tumour that recurs in the pelvis near the previous operative site [1-3]. Most, though not all, local recurrences manifest within two years of surgery. In this study local recurrence in the pelvis will be defined in three ways:

1. Definite recurrence – positive histology.

2. Probable recurrence – a postoperative mass in the area of previous pelvic surgery with any of the following patient signs (hydronephrosis, invasion of pelvic structures, or bleeding rectal mass) or symptoms (deteriorating sexual function, deteriorating bladder function, deteriorating bowel function, or persistent and worsening lower back, perineal or sciatic pain).

3. Possible recurrence – based strictly on a patient developing any of the symptoms mentioned above (deteriorating sexual function, deteriorating bladder function, deteriorating bowel function, or persistent and worsening lower back, perineal or sciatic pain).

#### Secondary outcomes

Surgeons determined which of their respective patients may be candidates to participate in secondary outcomes data collection. Surgeons introduced this part of the study to appropriate patients and if the individual was agreeable, the study team approached the respective patient to obtain written informed consent. Patients were not approached if they were too ill, frail or psychologically vulnerable, as determined by the participating surgeon. Patients required sufficient command of the English language and capacity to give informed consent. Secondary outcomes included:

#### Quality of life

To assess overall impact of disease and treatment on patient quality of life we used the following: the European Organization for Research and Treatment of Cancer (EORTC) QLQ-C30 [35], an instrument to assess quality of life in cancer patients; the supplemental EORTC QLQ-CR38 [36], a colorectal cancer-specific quality of life questionnaire module; and, the 36-Item Short Form Health Survey, Version 2 (SF-36) [37].

#### Bowel, bladder and sexual function

At the time of trial initiation we felt there was no validated instrument(s) that adequately addressed these three important domains. To this end a brief set of questions were developed [see Additional file 2].

### Sample size

We calculated the intracluster correlation coefficient using the a priori variation in permanent colostomy rates among hospitals in Ontario with a rectal cancer procedure volume of at least 15 per year for years 1997 to 1999 (Intracluster correlation = 0.4) [38,39]. We used a conservative spectrum of values for local recurrence intraclass correlation coefficients. Alpha and beta were set at 0.05 and 0.2, respectively. Tests were two-sided and assumed that a clinically important change would consist of decrease in the baseline rate of permanent colostomy from 30% to 15%. Using permanent colostomy as the outcome, the study required 8 hospitals and 311 patients in each arm. For local recurrence we extrapolated from the literature, and assumed that local recurrence rates are a conservative 20% and can be brought down to 8% in the intervention arm. To detect this difference with confidence we required 16 hospitals and 672 patients – 8 hospitals and 336 patients in each arm. Thus, the sample size required for the outcome local recurrence determined our final sample size requirements. We randomized 16 hospitals and anticipated an accrual period of 24 months.

### Randomization

The study statistician generated and administered a blocked 1:1 allocation randomization arrangement for the 16 study hospitals. We did not stratify hospitals by procedure-volume or teaching status since previous research in Ontario indicates that such hospital characteristics do not differentiate patient outcomes following colorectal cancer surgery [40,41]. Following the randomization of an intervention arm site, no further hospital was randomized until that site participated in a workshop (the first component of the QIRC strategy). This was done since patients were immediately accrued following site randomization, despite the typical requirement of 2–4 weeks to arrange a workshop with all or at least a majority of the surgeons at the respective site.

### Data gathering

Data are being collected for patients in the intervention and control groups in identical ways. Primary outcomes data are collected via chart review for at least 30 months, and thus follow-up will be greater for all but the final patients enrolled in the trial. This follow-up period will allow identification of local recurrence events, since local recurrence typically occurs within two years of surgery [1-3]. Hospital charts are reviewed within two weeks of surgery and every three months thereafter. Regional Cancer Centre charts are also reviewed to optimize the collection of data on potential patient treatments (e.g., radiation therapy) and outcomes (e.g., local recurrence). In Ontario, all radiation therapy is delivered at a small number of such Cancer Centres. Quality of life and bowel, bladder, and sexual function were measured at four times: baseline (pre-operatively or within 4 weeks of surgery) and at 6, 12 and 18 months post-surgery. Trained assessors obtained data for secondary outcomes during telephone interviews. All data will be handled with strict confidentiality, and study reports or presentations will maintain patient, surgeon, and hospital anonymity. Data collection will be complete by the end of 2007.

### Analysis

We will use the intention-to-treat principle to analyse data. We will use multiple imputation to handle missing data [42]. Treatment arms will be compared with respect to potential covariates using continuous and categorical univariable analyses. This will include patient (age, sex, comorbidities, weight and height), treatment (adjuvant therapies), tumour (stage, node counts, margin status, other histology measures), and hospital (teaching status, procedure volume) level variables. Methods of analysis, including adjustment for covariates, will use the cluster randomization trial design [38,39]. Specifically, the primary outcomes of colostomy rate and local recurrence rate will be assessed using a nested (cluster) analysis model that will consider the correlation of outcomes within each hospital, and the influence of covariables. Adjustments for adjuvant therapies are especially important since it is likely these treatments will not be delivered among centers using a standard regimen. The major design factor will be arm of study (experimental or control). No adjustment will be made for multiple comparisons of primary outcomes since each is of interest on its own. Secondary analyses will be conducted on bowel, bladder and sexual function, and quality of life using nested analysis of variance. We will conduct analyses using the latest versions of SAS, SPlus and StatXact software as needed. The trial will be reported according to the CONSORT standards for reporting cluster-randomized trials [43]. The results will be expressed as odds ratio [OR] (for binary outcomes), hazard ratio [HR] (for time-to-event outcomes) or mean difference (for continuous outcomes), with corresponding standard errors, 95% confidence intervals and associated p-values. P-values will be reported to three decimal places with p-values less than 0.001 reported as p < 0.001. For all tests, we will use alpha = 0.05 level of significance. We will examine residuals to assess goodness-of-fit and model assumptions for all analyses as appropriate.

### Nested studies

#### Surgeon survey

At the completion of accrual surgeons were mailed a survey. Intervention arm surgeons were asked to comment on their involvement in the trial, to evaluate the individual QIRC strategy interventions and the overall strategy, and to provide insights on issues that may be relevant to the uptake of a new innovation such as the QIRC strategy. A similar survey was sent to control arm surgeons, however, they were not asked to evaluate the individual QIRC strategy interventions. Participation in the survey was voluntary. Surgeons who agreed to participate were given the option to identify themselves or remain anonymous.

#### Qualitative interviews

Qualitative telephone interviews were conducted with opinion leaders in the intervention arm hospitals. At least one other surgeon from each of the intervention arm hospitals was also interviewed. Participation in the interviews was voluntary. The interviews will assist in assessing the role of the local opinion leader and the overall QIRC strategy

### Ethics

The study is being conducted according to the established guidelines of proper conduct of medical research involving human subjects set by the Tri-council Policy Statement and the GCP guidelines [44-48]. The Research Ethics Board (REB) of the Hamilton Health Sciences/McMaster University approved this protocol (REB project number 01-202). Each participating hospital also provided local ethics approval prior to enrollment. Ethics approval from Ontario Regional Cancer Centres was also necessary for the examination of study patients' charts. Individual surgeons provided written consent, as did all patients who participated in the collection of secondary outcomes data (i.e., quality of life scores, and questionnaires on bowel, bladder and sexual function).

## Discussion

The QIRC trial will assess if the QIRC strategy, which is designed to positively influence surgeon practice at a given hospital, can lead to improved patient outcomes of rectal cancer surgery. Lower permanent colostomy and local tumour recurrence rates in the intervention arm would suggest that the QIRC strategy is efficacious. The strategy may also act as a template for efforts to improve surgical quality in other cancer sites.

Participants in a clinical trial may alter – improve – their behaviour in response to being observed rather than as a result of a study intervention [49,50]. This phenomenon is known as the Hawthorne effect and it is important to consider in trials designed to influence physician behaviour and action. Surgeons in both arms of the QIRC trial may be subject to a potential Hawthorne effect. Surgeons in the control arm will consent to randomization and be cognizant of ongoing data abstraction. In addition to these maneuvers, surgeons in the experimental arm will receive the QIRC strategy. Thus, all surgeons in the study will be aware their actions are being observed. Any improvement in patient outcomes in the experimental arm must be above and beyond the Hawthorne effect, and would be attributed to the intervention.

We are aware of one other trial that tested if knowledge translation or quality improvement methods could influence (i.e., improve) surgeon practice [51]. This trial used continuing medical education and opinion leaders to improve (i.e., lower) rates of cesarean section. The relatively small number of randomized trials that involve surgeons usually compare the delivery of contrasting treatments (e.g., surgical versus medical therapy for patients with carotid stenosis, or, different methods of surgically treating patients with breast cancer). Thus, regardless of our results, successful engagement of surgeon participation in this cluster-randomized trial will be an important observation in and of itself. This trial does not compare total mesorectal excision-type surgery versus standard surgery, rather it is comparing a strategy designed to influence surgeon practice versus the normal environment. Thus, the trial will contribute greatly to knowledge on optimizing surgeon practice.

## Competing interests

The author(s) declare that they have no competing interests.

## Authors' contributions

The study was initiated by MS and ML. MS, CG, LT, RM, TW and ML are grant holders. All authors participated in the study concept and design. CG, LT and ML provided expertise in cluster randomized controlled trials. CG and LT provided statistical expertise. MS, RM and FD provided expertise in the surgical and pathologic treatment of rectal cancer. All authors reviewed and approved the final version of this manuscript.

## Pre-publication history

The pre-publication history for this paper can be accessed here:



## Supplementary Material

Additional file 1 Operative Questionnaire. Surgeons in the experimental arm completed this questionnaire after each rectal cancer surgery. The questions were designed to prompt surgeons to re-examine key total mesorectal excision operative steps. Click here for file

Additional file 2 Bowel, Bladder and Sexual Function Questions. Questions developed for the QIRC trial to address bowel, bladder and sexual function following rectal cancer surgery. Click here for file
