# Supplementary material for: The Quality Initiative in Rectal Cancer (QIRC) trial: study protocol of a cluster randomized controlled trial in surgery
Source: BMC Surg. 2008 Feb 15;8:4. doi: 10.1186/1471-2482-8-4 (PMC2262058; doi:10.1186/1471-2482-8-4)
Supplement: Additional file 1 — Operative Questionnaire. Surgeons in the experimental arm completed this questionnaire after each rectal cancer surgery. The questions were designed to prompt surgeons to re-examine key total mesorectal excision operative steps. [file 1471-2482-8-4-S1.doc]

# Quality Initiative in Rectal Cancer Trial

**Operative Questionnaire**

1a. Distance of distal edge of tumour from anal verge (ideally measured using rigid sigmoidoscopy):

_____ cm or ❒ Distance unknown

1b. Tumour at or below sacral promontory: ❒ Yes ❒ No ❒ Unsure

1c. Mark the location of the tumour on the diagram below.


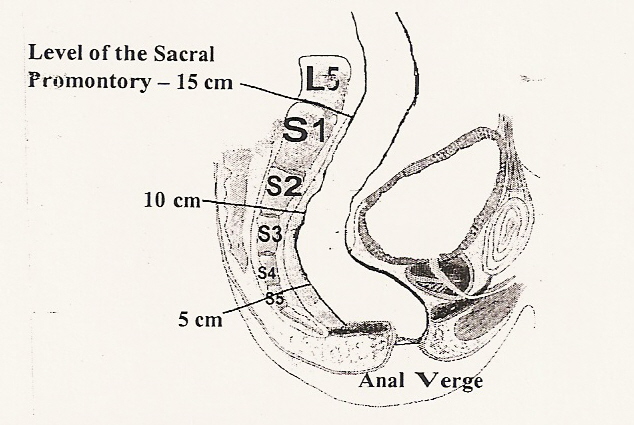


1. Circumferential location: (check all that apply)

❒ Anterior ❒ Posterior

❒ Left ❒ Right

3. Tumour penetration: ❒ Mobile ❒ Tethered ❒ Fixed

4. Local invasion: (check all that apply)

❒ Small bowel

❒ Large bowel

❒ Bladder

❒ Prostate

❒ Uterus

❒ Vagina

❒ Pelvic side wall

❒ Sacrum

❒ Other: __________________________________________

5. Procedure performed:

❒ Low anterior resection (LAR)

❒ Abdominoperineal resection (APR)

❒ Pelvic exenteration

❒ Hartman’s resection

6a) Were you satisfied with the dissection of the left paracolic gutter

and the division of the inferior mesenteric artery or descending branch? ❒ Yes ❒ No

b) Were you satisfied with the dissection of the mesorectum posteriorly? ❒ Yes ❒ No

c) Were you satisfied with the dissection of the mesorectum laterally? ❒ Yes ❒ No

d) Were you satisfied with the dissection of the mesorectum anteriorly? ❒ Yes ❒ No

e) Were you satisfied with the identification and preservation of

sympathetic nerves? ❒ Yes ❒ No

f) Were you satisfied with the preservation of the parasympathetic nerves? ❒ Yes ❒ No

g) Were you satisfied with the overall dissection of mesorectum? ❒ Yes ❒ No

h) Were you satisfied with the use of the sphincter sparing procedure? ❒ Yes ❒ No

7a. Did you inspect the rectal specimen? ❒ Yes ❒ No

7b. If so, were you satisfied with the overall appearance of the specimen? ❒ Yes ❒ No

8a. Was the surgery curative? ❒ Yes ❒ No

8b. If not, this was due to: ❒ Distant disease (liver, lung, peritoneal, etc..)

❒ Gross residual pelvic disease

❒ Microscopic residual pelvic disease- suspected or known

❒ Other (please specify): ______________________

9a. Did an operative demonstrator from the Quality Initiative in Rectal Cancer Trial assist you with this case?

❒ Yes ❒ No

9b. If an operative demonstrator was present for this case, was the operation in any way different compared to your traditional approach to rectal cancer surgery?

❒ Yes ❒ No

9c. If so, please indicate which aspect(s) of the operation were in any way different from your traditional approach.

- The method used to dissect the left paracolic gutter and the division of the inferior mesenteric artery or descending branch.
- The method used to dissect the mesorectum posteriorly.
- The method used to dissect the mesorectum laterally.
- The method used to dissect the mesorectum anteriorly.
- The method used to identify and preserve the sympathetic nerves.
- The method used to identify and preserve the parasympathetic nerve.
- Anastomosis was closer to the anal verge.
- Inspection of the rectal specimen.
- Use of diverting ileostomy.
- Use of sphincter sparing procedure.
- Other differences (please describe): ____________________________

10a. Will the operative demonstration change your intraoperative approach to rectal cancer surgery?

❒ Yes ❒ No ❒ Undecided

10b. Please comment:
