# Supplementary material for: The Quality Initiative in Rectal Cancer (QIRC) trial: study protocol of a cluster randomized controlled trial in surgery
Source: BMC Surg. 2008 Feb 15;8:4. doi: 10.1186/1471-2482-8-4 (PMC2262058; doi:10.1186/1471-2482-8-4)
Supplement: Additional file 2 — Bowel, Bladder and Sexual Function Questions. Questions developed for the QIRC trial to address bowel, bladder and sexual function following rectal cancer surgery. [file 1471-2482-8-4-S2.doc]

**Administration:**

Pre-surgery/baseline □

6 months post-surgery □

12 months post-surgery □

18 months post surgery □

**Date**: ______/________/_____ (dd/mmm/yy)

# Quality Initiative in Rectal Cancer Trial

**Bowel, Bladder and Sexual Function Questions**

**Bladder Function**

**1. Which of the following best describes your urine function TODAY:**

You are fully continent 1

You need to wear a pad 2

You must self catheterize 3

You have an indwelling catheter 4

Refused 8

Unsure/Unknown 9

**2. Overall, how would you rate your urine function?**

Excellent 1

Good 2

Fair 3

Poor 4

Refused 8

Unsure/Unknown 9

**Bowel Function**

**3. During the past WEEK, how many bowel movements did you have on an average day? ___________**

record number per day

**4. During the past WEEK, did you ever wear a protective pad or diaper during the day because of stool incontinence?**

Yes 1

No 2

Refused 8

Unsure/Unknown 9

1. **During the past WEEK, did you ever wear a protective pad or diaper at night because of stool incontinence?**

Yes 1

No 2

Refused 8

Unsure/Unknown 9

**6. During the past WEEK, did you avoid any social activities such as visiting friends, car trips, or going to the movies due to concerns about your bowel function?**

Always 1

Often 2

Sometimes 3

Never 4

Refused 8

Unsure/Unknown 9

**7. Overall, how would you rate your bowel function?**

Excellent 1

Good 2

Fair 3

Poor 4

Refused 8

Unsure/Unknown 9

The last set of questions asks you to tell us about any changes that you have noticed in your bowel, bladder and sexual function during the last 3 months.

**8. Over the past 3 MONTHS, have you:**

|  | **Yes**  **1** | **No**  **2** | **Refused**  **8** | **Unsure/Unknown**  **9** |
| --- | --- | --- | --- | --- |
| Noticed any persistent and worsening lower back pain | 1 | 2 | 8 | 9 |
| Noticed any new pain down the back of your legs | 1 | 2 | 8 | 9 |
| Noticed any new pain around your anus | 1 | 2 | 8 | 9 |
| Noticed a worsening of your bowel function | 1 | 2 | 8 | 9 |
| Noticed a worsening of your urine function | 1 | 2 | 8 | 9 |
| Noticed a worsening of your sexual function | 1 | 2 | 8 | 9 |
